# Biofunctionalised bacterial cellulose scaffold supports the patterning and expansion of human embryonic stem cell-derived dopaminergic progenitor cells

**DOI:** 10.1186/s13287-021-02639-5

**Published:** 2021-11-13

**Authors:** Miranda Robbins, Venkat Pisupati, Roberta Azzarelli, Samer I. Nehme, Roger A. Barker, Ljiljana Fruk, Gabriele S. Kaminski Schierle

**Affiliations:** 1 Department of Chemical Engineering and Biotechnology, University of Cambridge, West Cambridge Site, Philippa Fawcett Drive, Cambridge, CB3 0AS UK; 2 John Van Geest Centre for Brain Repair and WT-MRC Cambridge Stem Cell Institute, University of Cambridge, Cambridge, CB2 0PY UK; 3 Unit of Cell and Developmental Biology, Department of Biology, University of Pisa, S.S. 12 Abetone e Brennero 4, 56127 Pisa, Italy; 4 Wellcome - MRC Cambridge Stem Cell Institute, Jeffrey Cheah Biomedical Centre, Cambridge Biomedical Campus, University of Cambridge, Cambridge, CB2 0AW UK

**Keywords:** Stem cells, Neurodegeneration, Biofunctionalisation, Cell scaffold, Implantation, Tissue engineering

## Abstract

**Background:**

Stem cell-based therapies for neurodegenerative diseases like Parkinson’s disease are a promising approach in regenerative medicine and are now moving towards early stage clinical trials. However, a number of challenges remain including the ability to grow stem cells in vitro on a 3-dimensional scaffold, as well as their loss, by leakage or cell death, post-implantation. These issues could, however, be helped through the use of scaffolds that support the growth and differentiation of stem cells both in vitro and in vivo. The present study focuses on the use of bacterial cellulose as an in vitro scaffold to promote the growth of different stem cell-derived cell types. Bacterial cellulose was used because of its remarkable properties such as its wettability, ability to retain water and low stiffness, all of which is similar to that found in brain tissue.

**Methods:**

We cultured human embryonic stem cell-derived progenitor cells on bacterial cellulose with growth factors that were covalently functionalised to the surface via silanisation. Epifluorescence microscopy and immunofluorescence were used to detect the differentiation of stem cells into dopaminergic ventral midbrain progenitor cells. We then quantified the proportion of cells that differentiated into progenitor cells and compared the effect of growing cells on biofunctionalised cellulose versus standard cellulose.

**Results:**

We show that the covalent functionalisation of bacterial cellulose sheets with bioactive peptides improves the growth and differentiation of human pluripotent stem cells into dopaminergic neuronal progenitors.

**Conclusions:**

This study suggests that the biocompatible material, bacterial cellulose, has potential applications in cell therapy approaches as a means to repair damage to the central nervous system, such as in Parkinson’s disease but also in tissue engineering.

## Introduction

One major area of regenerative medicine includes the use of stem cells, progenitor cells or tissue transplants to restore the physiological function of diseased tissue through either (1) direct cell replacement, (2) by providing trophic support to the remaining host cells and matrix or (3) by modulating aspects of the local environment such as the immune response to the diseased tissue [30]. Early stage clinical trials are currently emerging in the field of neurodegeneration [27, 28, 44]. For example, in Parkinson’s disease (PD), trials are under way to use stem cells to replace lost nigral dopaminergic neurons that lie at the heart of the symptoms observed in PD patients [15, 32, 33]. In these trials, PD patients receive transplants of human foetal ventral mesencephalic cells into the striatum as a means to replace the lost dopaminergic innervation of the striatum which can lead to long-lasting relief of clinical features of PD patients [13, 27, 28, 30, 48]. However, the use of foetal tissue is associated with major logistical and ethical problems which may be solved by using human pluripotent stem cells instead. Human pluripotent stem cells are extracted from left-over early-stage embryos following infertility treatment which, after informed consent, can be donated to medical research [34].

Human embryonic stem cells (hESCs) are pluripotent stem cells derived from the inner cell mass of blastocysts and have the ability to differentiate into different cell lineages under appropriate culture conditions. Protocols have been developed to differentiate ESCs into precursors of multiple cell types including dopaminergic neurons affected in PD [25, 37]. However, despite these successes there are several key challenges in taking such therapies forward to larger-scale clinical trials including their directed differentiation and survival pre- and post-implantation [30]. One strategy to try and better support transplanted cells would be through the use of an implantable biodegradable scaffold that aids adhesion of cells.

Cell scaffolds provide a three-dimensional biomimetic environment that supports the culture and growth of cells and tissue; ideally, these scaffolds should (1) contain a highly porous and permeable matrix in which nutrients and metabolic waste can be exchanged during cell growth, (2) be biocompatible, biodegradable and chemically tuneable to permit surface modification to guide cell adhesion and differentiation and (3) have mechanical properties that closely match the tissue at the site of implantation in order to increase cell integration and to decrease the chance of tissue rejection [14].

**Fig. 1:**
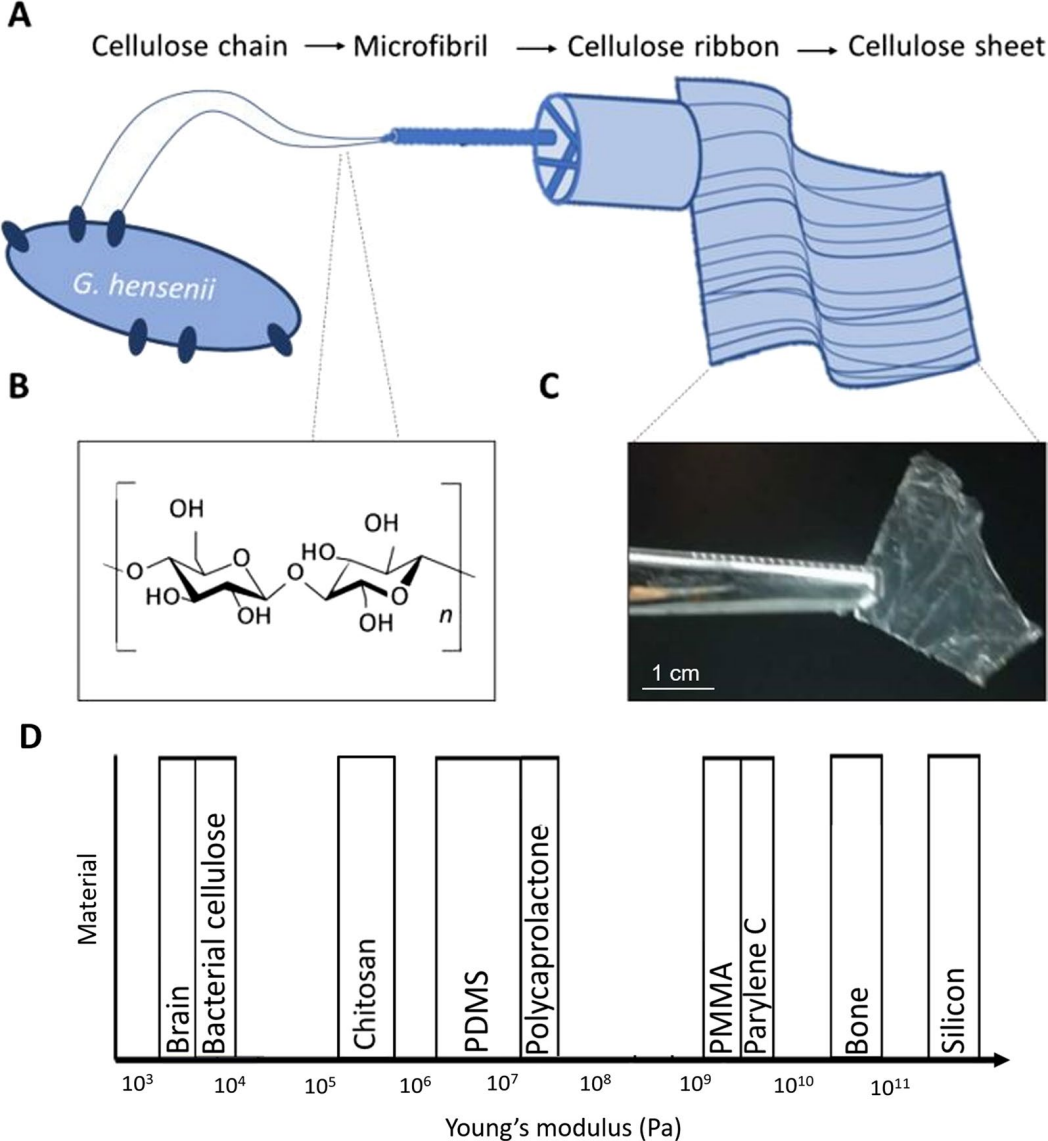
Bacterial cellulose sheets are made by cellulose chains produced by bacteria. **A** Bacterial cellulose initially forms cellulose chains that through hydrogen bonding turn into microfibrils which consequently form larger, well-spaced ribbons/nanofibres and ultimately cellulose sheets. **B** The repeated monomeric structure of cellulose then forms a cellulose chain. **C** A piece of freeze-dried cellulose sheet after overnight dehydration. **D** BC is most similar in stiffness to the brain as compared to other commonly used biomaterials for fabrication of medical devices. Furthermore, BC is highly biocompatible and therefore is an optimal material for the fabrication of prosthetics that require moulding to brain regions

Numerous implantable scaffolds have been investigated in order to match the mechanical and physiological properties of the local tissue. Chitosan, a biopolymer made by alkaline *N*-deacetylation of chitin, has also been used as a neural scaffold due to its biocompatibility, low toxicity and low cost [9]. However, as a material, chitosan, with a Young’s modulus of 3–10 kPa, is far more rigid than brain tissue [47], with a Young’s modulus of 0.2–0.8 MPa (Fig. 1D)[8]. The same is true for PDMS (polydimethylsiloxane), a material commonly used for in vivo microfluidic cell devices, which has been used successfully in 3D cultures of primary hippocampal neurons [26]. However, besides being stiffer than brain tissue (10: 1 base to crosslinker, 580 kPa), PDMS also requires additional coating to reduce its hydrophobicity [38]. Thus, as the tools and techniques for stem cell differentiation and cell growth improve, there is a continuous need for matrix material that can act as a cellular scaffold and matches the material properties of the surrounding brain tissue. Herewith, we describe the use of bacterial cellulose as a suitable cell scaffold material.

Cellulose is the most abundant biopolymer in nature, and bacterial cellulose (BC), in particular, has attracted much interest in the material science community as it is produced using ecologically sustainable strategies and has several advantages over plant cellulose. Although BC and plant cellulose have the same chemical structure of (C_6_H_10_O_5_)_*n*_, BC has higher purity, and its well-spaced nano- and microfibrils result in higher strength, mouldability and increased water capturing ability compared to plant cellulose [11, 19]. The *Gluconacetobacter hansenii* bacterial strain is most widely used for BC production due to its high yield. These bacteria often produce cellulose as a part of a stress response, because in the absence of oxygen, BC is usually produced as a 1% w/v thin film that rises to the air–liquid interface carrying aerobic bacteria as the source of oxygen [24, 45].

The unique cellulose structure is produced when hundreds-to-thousands of cellulose chains are extruded from pores on the bacterial cell envelope. These chains first combine into microfibrils through hydrogen bonding and further into cellulose ribbons that make up the macrostructure of cellulose pellicles [45]. A cellulose sheet, which is a well-spaced 3D mesh of ribbons, is highly porous (Fig. 1A). These cellulose sheets can be dehydrated by freeze-drying to improve the shelf life and for further modification (Fig. 1C).

The biocompatibility, vapour permeability, moulding ability, structural integrity and high water capturing capability make BC highly suitable for medical applications such as wound dressing, tissue engineering and guided tissue regeneration [10]. Furthermore, ribbons of bacterial cellulose microfibrils are structurally similar to native extracellular matrices produced by mammalian cells [11]. In addition, BC is composed of nearly 100% cellulose only and thus does not contain any additional polymeric and small molecule components, making it easier to purify and characterise unlike many other biopolymers including plant-based cellulose [32, 33]. Both the structural arrangement of BC and the absence of other molecular components may thus explain why BC has not been found to evoke an immune response from in vitro and in vivo studies [16, 22, 23, 35].

For tissue engineering and regeneration, cell scaffolds are often used to support cell integration by providing an interface for cells whilst they form cell–cell connections and their own extracellular matrix. The use of BC has already been shown to provide a monolayer of fibroblasts and/or keratinocytes that can be directly placed into wounds to promote tissue regeneration. In these cases, BC is soaked in serum and electrolytic solutions such as sodium hydroxide or adsorbed in collagen to promote cell adhesion [10, 51].

Various composites of BC and other materials such as graphene have also been assembled to enable stem cell differentiation into various cell lines including neurons. Particularly exciting is the use of BC scaffolds for the growth of neurons [26]. The ability to grow neurons on BC cellulose is highly advantageous due to its increased biocompatibility and reduced rigidity. Alongside this, BC has a similar stiffness to brain, of around 7 kPa [36], and more robust mechanical properties and thermostability compared to other naturally derived polymers including matrigel, collagen and silk (Fig. 1D) [52].

In addition to its inherent properties, additional functionalities can be introduced by BC modification, for example, by activation of bioactive peptides or small molecules. The biofunctionalisation of stem cell scaffolds using growth factors (GFs) has previously been shown to enhance stem cell differentiation or improve their functionality [5, 22, 23, 39]. The presence of GFs within the scaffold removes the need for further injection of GFs after the cells have been implanted and also circumvents the challenge of getting such factors across the brain–blood barrier.

In order to design a functional and biocompatible stem cell and neuronal scaffold, we have investigated bacterial cellulose covalently modified with growth factors BDNF (brain-derived neurotrophic factor) and GDNF (glial cell-derived neurotrophic factor). Directed differentiation of progenitor cells using BDNF has been previously investigated [7, 18, 20, 31, 40, 54]. Recently, Horne et al. used aminolysation of BDNF onto nanofibre scaffolds as a means to direct progenitor cells towards differentiation into oligodendrocytes and neurons [18]. However, they employed a polycaprolactone polymer, which degrades slowly over time, has poor cell adhesion, wettability and mechanical properties with a stiffness of 50 MPa [53]. The latter is more commonly used for bone tissue engineering, making it less suitable for softer brain tissue implantation. GDNF is another neurotrophic factor that has been shown to improve the survival and plasticity of dopaminergic neurons in vitro and following transplantation [2, 12, 21, 29, 49]. GDNF delivery through hydrogel scaffolds has previously been shown to increase regeneration and nerve repair [46], and pre-treatment of progenitor cells with GDNF improves cell survival following transplantation in a rodent Parkinson’s disease model [2, 6].

We therefore sought to investigate the use of BDNF- and GDNF-modified BC sheets as a scaffold for neuronal progenitor cells. As a first step, we showed that the adhesion of ES-derived progenitor cells to the cellulose membrane was sufficient to ensure sufficient growth, as this has not previously been shown for neural progenitor cells. As a next step, we showed that a biofunctionalisation method previously used on cellulose nanoparticles could also be used optimally for use on cellulose sheets. This functionalisation method was then used to covalently bind the growth factors, BDNF and GDNF to BC. Our results show that the functionalisation of BDNF and GDNF increases the number of cells that grow and differentiate on functionalised cellulose scaffolds into dopaminergic neuronal progenitors expressing LMX1 (LIM-homeodomain 1) and FOXA2 (Forkhead box protein).

Overall, this work presents a first step in the development of biofunctionalised bacterial cellulose as a cell scaffold for potential use in cell replacement therapies, such as neural grafting of human embryonic stem cell-derived dopaminergic neurons in Parkinson’s disease patients.

## Methods

### The production of bacterial cellulose

The wildtype bacterium strain *Gluconacetobacter hensenii* was used following the method described by Hestrin and Schramm [17]. For the preparation of bacterial cultures, 500 mL Hestrin and Schramm (H–S) medium was made in H_2_O containing (w/v): 0.5% yeast extract, 0.5% peptone, 0.27% Na_2_HPO_4_ dibasic, 0.15% citric acid and 2% glucose (added following autoclaving), at pH 6. For culturing in liquid solution, 5 mL of H–S medium was added to a 50-mL falcon tube; triplicates of *G. hensenii* culture were selected and kept at 30 °C for 7 days. For cellulose production, 100 µL of bacterial culture was added to a petri dish of 5 mL H–S medium and incubated at 30 °C for 2 weeks. For cleaning, cellulose was removed from the cell solution and washed several times with dH_2_O. Cellulose was then purified with 0.1–1 M NaOH at 70 °C for 2 h during which the cellulose changed colour to off-white/colourless. Cellulose was washed with dH_2_O until the pH reached pH 7. The cellulose was freeze-dried for longer-term storage and kept in dH_2_O or PBS [43].

### The preparation and biofunctionalisation of bacterial cellulose

In this study, we used freeze-dried macrosheets instead of the never-dried or the nanofibre forms of BC.

The ‘conventional’ method for the silanisation of cellulose fibres described by Salon et al. [42] is composed of two steps, hydrolysis and condensation. (1) Hydrolysis of 5% (3-Glycidyloxypropyl)trimethoxysilane (GPTMS, > 98%, Sigma-Aldrich, St. Louis, USA) was achieved in 80:20 v/v isopropanol:water for 2 h at room temperature. After 10 min, the cellulose was added, and the mixture was placed on a shaker. This permitted the adsorption of the silanol through acid–base bonding to the hydroxyl groups of cellulose. (2) Chemical condensation was ensured by heating to 120 °C using a hot plate until dry (~ 5 min) and results in the formation of siloxane bridges. The protein used for functionalisation, in this case Alexa Fluor® 488-conjugated goat anti-rabbit IgG (ab150077, Abcam, Cambridge, UK), can then be placed in contact with the dried, cooled cellulose surface for ~ 4 min before washing 3 × in PBS.

The ‘aqueous one-pot silanisation’ method was first described by Beaumont et al. [3, 4]. In this method, cellulose is placed in H_2_O and acidified with 1% v/v 0.5 M HCl to achieve the hydrolysis. To this, 0.5% v/v GPTMS (> 98%, Sigma-Aldrich) is added and shaken for 30 min. Following this initial treatment, 2% v/v 0.5 M NaOH is added for a further 2 h to induce the condensation. Cellulose is removed and washed 1 × with acetone and briefly dried under nitrogen before the addition of a model protein, Alexa Fluor® 488-conjugated goat anti-rabbit IgG (ab150077, Abcam). After ~ 5 min, excess protein is removed from the cellulose by washing it 3 × with PBS.

Following the procedure for the aqueous one-pot silanisation method, laminin is added to cellulose at 2 µg/mL concentration in Hanks' Balanced Salt Solution (HBSS; Sigma-Aldrich). In addition to laminin, cellulose sheets were biofunctionalised with growth factors BDNF (Sigma-Aldrich) at 8 µg/mL and GDNF (Sigma-Aldrich) at 4 µg/mL. To determine whether the functionalisation method affects the cell growth, 20 µg/mL laminin (Sigma-Aldrich) was added to plastic wells (96 multiwell plate, Corning®, Sigma-Aldrich) containing bacterial cellulose, without the biofunctionalisation step. All samples are incubated overnight before washing 3x ~ 5 min with HBSS.

### Culturing and plating of human embryonic stem cell-derived progenitor cells

RC17 (Roslin Cells, cat. no. hPSCreg RCe021-A) cells were maintained in iPS brew XF (Miltenyi, Bergisch Gladbach, Germany) on laminin-521 (0.5 µg/cm^2^ Biolamina LN-521)-coated plates and were patterned towards a ventral midbrain fate as described in Nolbrant et al. [37]. In brief, differentiation of hESCs to dopaminergic progenitors is a 16-day protocol. For neuralisation, the hES cell colonies were detached with EDTA (0.5 µM/ml) and plated at 10,000 cells/cm^2^ on laminin (Sigma-Aldrich)-coated plates in N2 medium (DMEM/F12:Neurobasal (1:1), N2 supplement (1:100), ROCK inhibitor (Y-27632, 10 µM, Tocris Bioscience, Bristol, UK), SB431542 (10 µM, Tocris Bioscience) and Noggin (100 ng/ml, R&D), and for patterning to ventral mesencephalic fate, SHH-C24II (300 ng/ml, Miltenyi) and 0.85 µM CHIR99021(Miltenyi) were added to the medium from day 0–9. On day 9, the cells were grown in N2 medium supplemented with FGF8b (100 ng/ml, Miltenyi). On day 11 of differentiation, the cells were dissociated to single cells with accutase (75 µl/ cm^2^, Thermo Fisher) and replated onto lam-111 coated/bacterial cellulose plates at 800,000 cells/cm^2^ in B27 medium (Neurobasal, B27 supplement without vitamin A (1:50), brain-derived neurotrophic factor (BDNF) (20 ng/ml, Miltenyi), glial cell line-derived neurotrophic factor (GDNF) (10 ng/ml, Miltenyi), FGF8b and ascorbic acid (200 mM, Tocris Bioscience). Cells grown on functionalised bacterial cellulose contained B27 medium with FGF8b and ascorbic acid alone.

### MTS cell proliferation assay

The MTS cell proliferation assay was based on the instructions provided (CellTiter 96® AQ_ueous_ One Solution Cell Proliferation Assay (MTS), Promega, Madison, USA). In brief, 20 µL MTS solution was added to DIV3 (days in vitro) cells in 100 µL of culture medium and incubated for 1 h at 37 °C at 5% CO_2_. Absorbance at 490 nm was measured using a 96 multiwell plate reader (Envision 2104 Multilabel Reader, PerkinElmer, Waltham, USA).

### Imaging and quantifying the growth of neural stem cells

Cells were fixed in 4% paraformaldehyde for 10 min at room temperature and washed 3 × in 37 °C PBS. Afterwards, the cells were incubated in blocking solution composed of PBS, 0.2% Triton-X and 4% donkey serum for 1 h at 4 °C. Primary antibody, anti-FOXA2 (R&D Systems AF2400) and anti-LMX1 (Merck Millipore, AB10533) were added 1:100 into the blocking solution (PBS, 5% donkey serum, 0.1% Triton X10), and the cells were incubated for 2 h at room temperature. Samples were washed in 3 × blocking solution before the addition of 1:500 donkey anti-goat IgG secondary antibody, Alexa Fluor® 561 (Thermo Fisher Scientific, Waltham, USA) and 1:500 donkey anti-goat IgG (H + L) secondary antibody, Alexa Fluor® 647 (Thermo Fisher Scientific) to the cells for 1 h at room temperature. Samples were washed 3 × in blocking solution , and 1 × in PBS. 1:1000 Hoechst 33,342 (Invitrogen, Thermo Fisher Scientific) was added to the cells in PBS before imaging them.

Samples were imaged using a custom-made widefield microscope with an Olympus frame (IX83, Olympus, Tokyo, Japan) and automated stage (Prior, Fulbourn, UK), plasma light source (HPLS343, Thorlabs, Newton, USA) and camera (Clara interline CCD camera, Andor, Belfast, UK) with filter sets for DAPI (49,000–ET–DAPI, Chroma Technology, Bellows Falls, USA), Alexa Fluor® 561 (49,008–ET–mCherry, Texas Red®, Chroma Technology) and Alexa Fluor® 647 (676/29 nm emission filter, Semrock, Rochester, USA). Pre-processing of images included background subtraction and intensity thresholding in ImageJ [41]. Counting was performed manually.

### Statistical methods

Data visualisation and statistical analysis were performed with GraphPad Prism 6.0 software (Graphpad, La Jolla, USA). All numerical data are expressed as mean ± standard deviation (SD) unless stated otherwise. Results (n ≥ 3 in all cases, unless stated otherwise) were analysed by unpaired, two-tailed *t* tests for 2 experimental groups, or by one-way analysis of variance (ANOVA) with multiple comparisons for 3 + groups of data. *P* values < 0.05 were considered statistically significant.

## Results

### Bacterial cellulose can readily be functionalised with growth factors

**Fig. 2:**
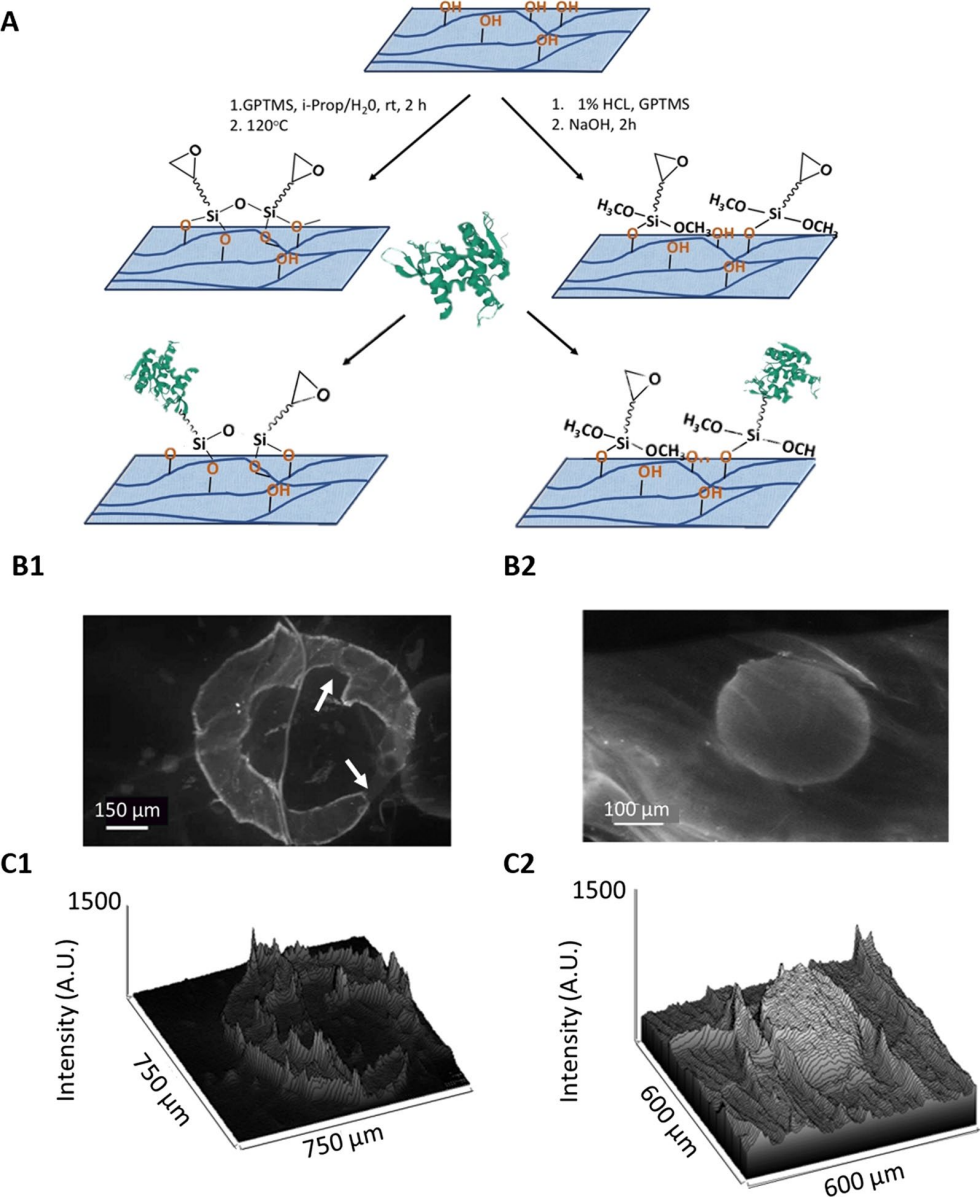
A comparison of two methods for the biofunctionalisation of bacterial cellulose with a model protein shows improved results for the ‘one pot’ method. **A** Both conventional (left) and aqueous one-pot (right) strategies were used to prepare glycido-coated cellulose through silanisation followed by covalent adherence of protein. AlexaFluor®-488-conjugated antibody immobilised onto the cellulose modified by (**B1**) conventional method results in cracking of protein, as shown by arrows but not in (**B2**) using the ‘aqueous one pot’ method. Surface fluorescence intensity profiles show an increase in intensity in regions where AlexaFluor®-488-conjugated antibody has covalently bound to cellulose (**C1**, **C2** representing conventional and aqueous one pot strategy, respectively). The fluorescence map was obtained by plotting the intensity profile of the conjugated antibody attached to BC

The conventional strategy uses 3-glycidopropyltrimethoxysilane (GPTMS) binding and high temperature curing, and the one-pot strategy is in aqueous conditions (Fig. 2A). Both resulted in glycido-functionalised cellulose, which can successfully be employed for protein immobilisation through amine groups. AlexaFluor®-488-labelled antibody was used as a model protein to assess the applicability of each strategy (Fig. 2B, C).

The fluorescence intensity profile across the functionalised area (Fig. 2C1, C2) shows a more uniform functionalisation of the protein in Fig. 2C2 as compared to in Fig. 2C1, where cracks in the protein are visible. As the functionalised scaffolds are inserted into the wet environment of the brain, it is important that the method is compatible with rehydration and there is no damage to the scaffold upon insertion. If scaffold breakage was to occur once the implant is inserted, this could result in an uneven distribution of the protein, clumps of composite material and even physiological microdamage, which might have significant negative consequences. For this reason, the ‘aqueous one-pot’ method was selected as a method of choice for further functionalisation of BC sheets and stem cell differentiation.

### Functionalised bacterial cellulose is fully biocompatible

**Fig. 3:**
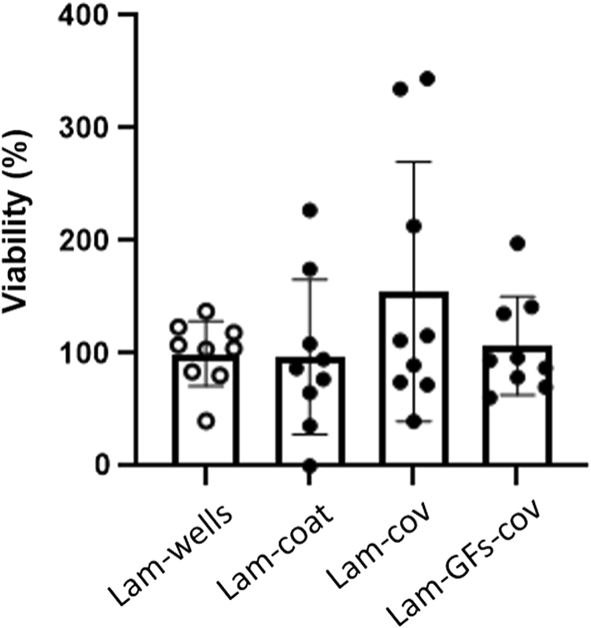
MTS assay shows there is no difference in the viability of hES-derived VM progenitor cells plated on plastic (open circles) or cellulose. Values represent absorbance at 490 nm as normalised to laminin coated plastic (Lam-wells). The functionalisation method requiring covalent binding of peptides to cellulose has no negative effects on the cell metabolism, as seen from MTS assay data obtained for functionalised laminin + BDNF + GDNF (Lam-GFs-cov) or functionalised laminin only (Lam-cov); one-way analysis of variance (ANOVA); *P* = 0.7883; *F* (3, 8) = 0.3532. Tukey’s multiple comparison P values (Laminin (A), cellulose + laminin (B), cellulose + functionalised laminin (C), cellulose + functionalised GFs (D): (A–B) not significant (NS) = 0.8655; (A–C) NS = 0.8634; (A–D) NS ≥ 0.9996; (B–C) NS ≥ 0.9999; (B–D) NS = 0.9041; (C–D) NS = 0.9023). Experiments were performed from *N* = 3 experimental repeats with *n* = 3 technical repeats. Mean values ± standard deviation are plotted

Following the optimisation of the functionalisation protocol using an antibody, BC was next functionalised with laminin alone or with laminin *and* the growth factors (GFs) BDNF and GDNF. These were employed with the aim to determine whether they support the proliferation and maturation of human embryonic stem (ES) cell-derived ventral midbrain (VM) progenitor cells [25, 37], and improve the growth of these cells on BC. Laminin is required for cell survival and was used in all tested samples. VM progenitor cells were plated on to laminin-coated 96 multiwell plates (Lam-wells), laminin-coated cellulose (Lam-coat), cellulose functionalised with laminin (Lam-cov), and cellulose functionalised with laminin and both of the growth factors, BDNF and GDNF (Lam-GFs-cov). Shown in Fig. 3 are MTS assays, a colorimetric assay for measuring cell metabolic activity, performed to assess the cell viability.

The results, as seen from MTS assay data obtained for functionalised laminin + BDNF + GDNF (Lam-GFs-cov) or functionalised laminin only (Lam-cov), show that there are no differences in the viability of hES-derived VM progenitor cells plated on different substrates, nor was the cell viability affected by the functionalisation of cellulose. Although the MTS assay clearly shows that bacterial cellulose does not affect the growth and survival of cells, it is important to determine whether it maintains the identity of VM progenitor cells.

### The cellulose scaffold supports the expression of dopaminergic transcription factors in VM progenitors

**Fig. 4:**
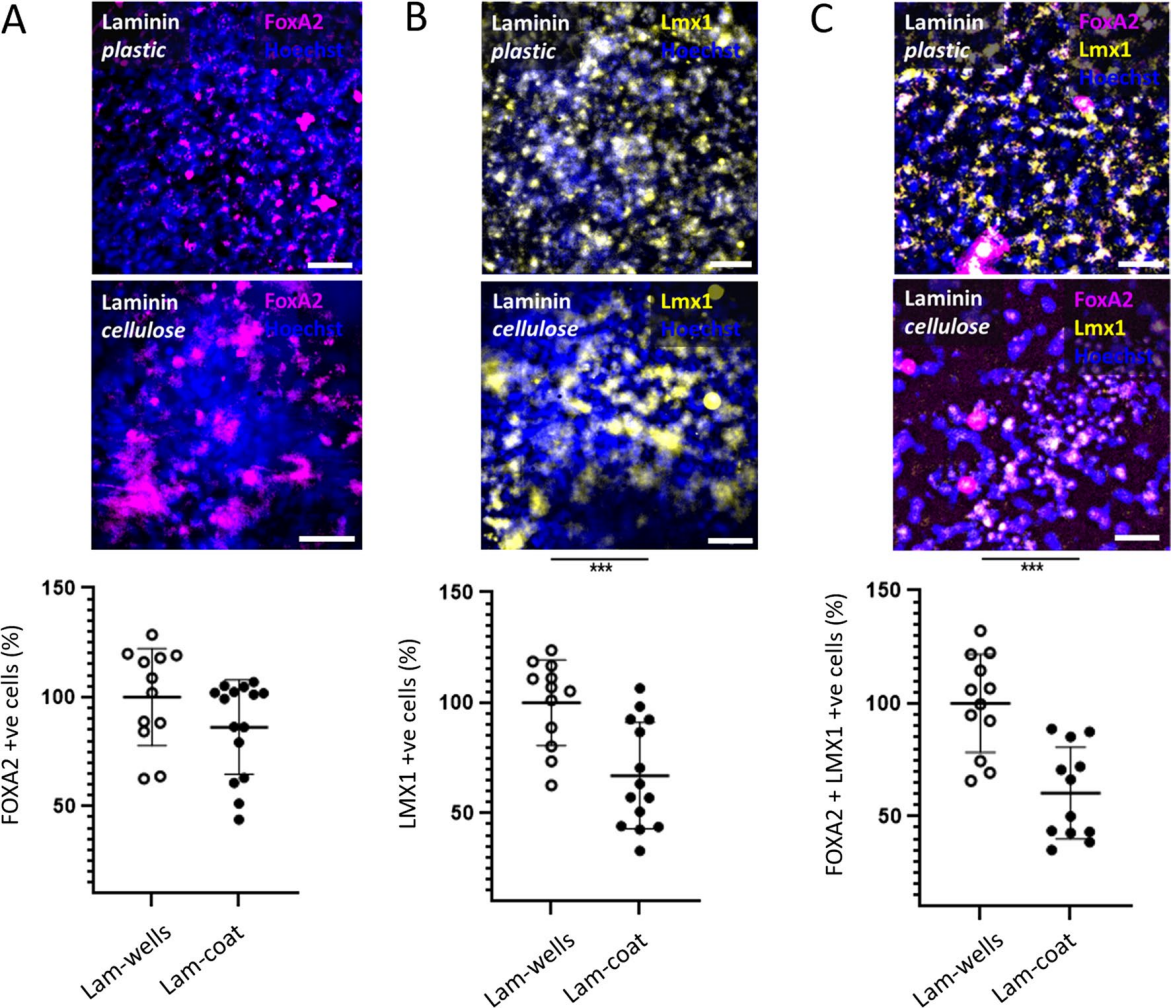
hES-derived VM progenitors grown on bacterial cellulose express markers of dopaminergic lineage. VM progenitors grown on laminin coated bacterial cellulose (Lam-coat) express FOXA2, LMX1, and FOXA2 and are colocalised with LMX1. The expression of FOXA2 is comparable to the plastic substrate controls (Lam-wells). The expression of LMX1 and LMX1 with FOXA2 is decreased compared with cells cultured on plastic. The proportion of cells showing colocalisation of a nuclear marker, Hoechst 33,342 (blue) with the transcription factor **A** FOXA2 (magenta); **B** LMX1 (yellow); **C** FOXA2 (magenta) and LMX1 (yellow). Two-tailed unpaired Student’s *t* test were used for statistical analysis. ***P < 0.001. Experiments were performed from *N* = 3 experimental repeats with *n* = 3 technical repeats. All data are plotted with mean values ± standard deviation normalised to Laminin coated plastic (Lam-wells). Scale bar: 30 µm

After we showed that culturing VM progenitors on bacterial cellulose did not affect their survival, experiments were performed to determine whether different scaffolds supported the caudalisation and proliferation of VM progenitor cells. The transcription factors FOXA2 (Fig. 4A) and LMX1 (Fig. 4B) were used as markers of VM progenitor identity. The proportion of cells expressing FOXA2 or LMX1 was calculated, alongside the proportion of cells expressing both transcription factors.

BC coated with laminin supports differentiation of stem cells to dopaminergic precursors as shown by FOXA2 and LMX1 expression. Expression of FOXA2 is similar in cells grown on plastic and BC. However, as shown in Fig. 4, there is a reduction in number of cells expressing LMX1 and LMX1 colocalised with FOXA2.

We were then interested to investigate whether the functionalisation of either laminin, or laminin with GFs would further support the growth and differentiation.

**Fig. 5:**
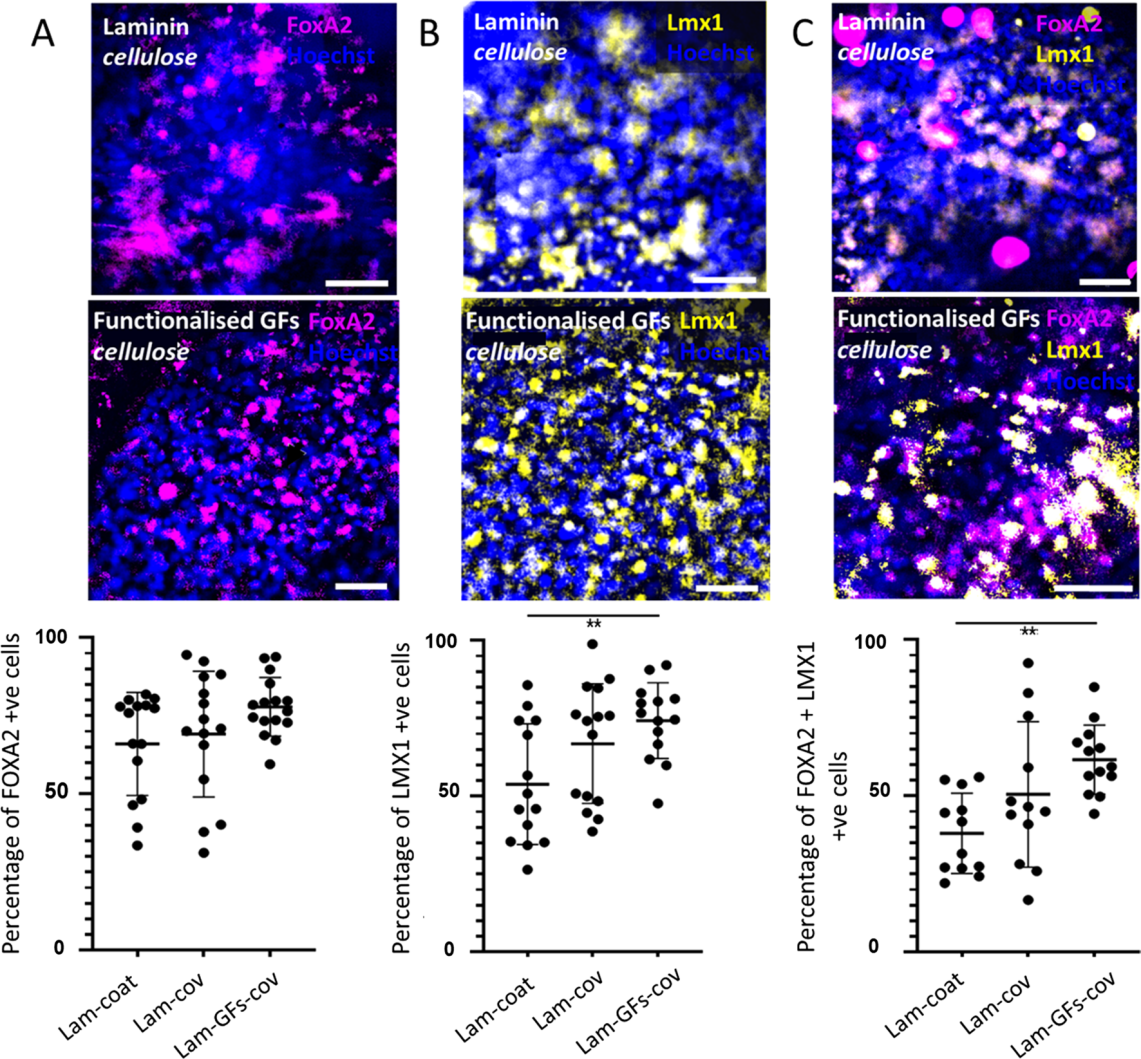
Growth of stem cells on GF-functionalised bacterial cellulose further increases the expression of FOXA2, LMX1, and FOXA2 colocalised with LMX1 compared to laminin only coated cellulose samples. The percentage of cells showing co-localisation of a nuclear marker, Hoechst 33,342 (blue) with the transcription factor **A** FOXA2 (magenta); **B** LMX1 (yellow); **C** FOXA2 (magenta) and LMX1 (yellow), as determined by manual counting. **A** (one-way analysis of variance (ANOVA); *P* = 0.1090; *F* (2, 43) = 2.145). Tukey’s multiple comparison *P* values [Lam-coat (**A**), Lam-cov (**B**), Lam-GFs-cov (**C**)]: **A**, **B** not significant (NS) = 0.8503; **A**–**C** NS = 0.1065; **B**, **C** NS = 0.2894. **B** (one-way analysis of variance (ANOVA); *P = 0.0116; F (2, 40) = 1.961) Tukey’s multiple comparison *P* values [Lam-coat (**A**), Lam-cov (**B**), Lam-GFs-cov (**C**)]: **A**, **B** not significant (NS) = 0.1200; **A**–**C** **P = 0.0093; **B**, **C** NS = 0.4905. **C** (one-way analysis of variance (ANOVA); **P = 0.0044; *F* (2, 34) = 2.152). Tukey’s multiple comparison *P* values [Lam-coat (**A**), Lam-cov (**B**), Lam-GFs-cov (**C**)]: **A**, **B** not significant (NS) = 0.1200; **A**–**C** **P = 0.0030; **B**, **C** NS = 0.2250

As shown in Fig. 5, the functionalisation of cellulose with laminin, BDNF and GDNF increases the number of cells that express the transcription factors LMX1 (Fig. 5B), and colocalised FOXA2 and LMX1 (Fig. 5C) in nuclei as compared with laminin–cellulose alone. This suggests that the biofunctionalisation of these growth factors increases the maturation of stem cells into the dopaminergic neuronal lineage.

However, the functionalisation of laminin alone does not increase the expression of FOXA2 and LMX1 significantly above that seen on the laminin-coated cellulose sample.

These results support our hypothesis that BC can be employed as a scaffold for differentiation of embryonic stem cells and that GF-functionalised BC further improves the expression of early dopaminergic markers in these cells.

## Discussion

In this work, we have optimised a method to biofunctionalise bacterial cellulose sheets with growth factors and use them as cell scaffold that may, once proven successful in vivo, become useful for the implantation of stem cells into patients. Studies on human embryonic stem cells (hESC) demonstrated that the BC scaffold supports differentiation of these stem cells to dopaminergic VM progenitor cells which are used for cell replacement therapies in Parkinson’s disease. The functionalisation of the scaffold with laminin, and growth factors BDNF and GDNF further increased the proportion of cells that differentiate once plated on BC. Such covalent attachment of GFs can prevent leakage of the protein and provide cells with long-term supply of GFs necessary for successful differentiation and growth of VM progenitor cells. In addition, covalent GF-functionalisation is also highly cost-effective as it reduces the concentration of growth factors that need to be exogenously added to the medium during cell culture.

Biofunctionalisation of cellulose was based on previously reported strategy employing salinisation of cellulose fibres and nanocellulose, but needed to be adapted to large sheets of freeze-dried BC [3, 4]. Previously developed silanisation methods of cellulose used ‘never-dried’ or nanofibre forms of cellulose, as opposed to freeze-dried macrosheets. We therefore tested two methods of silanisation, the ‘conventional’ and the ‘one-pot’ method to determine whether they could be used for freeze-dried cellulose. Although the conventional ‘curing’ method required fewer steps, it resulted in hornification, temporary cellulose shrinkage and crosslinking of the trivalent silanol. This in turn resulted in cracking of the functionalised protein layer during rehydration in PBS (Fig. 2B1) [1, 3, 4]. The conventional method is therefore not ideally suited for the preparation of uniformly coated and stable cellulose samples. In contrast, the ‘aqueous one-pot silanisation’ method [3, 4] does not lead to fractures in the protein layer following the full rehydration in PBS (Fig. 2B2).

Despite the current study highlighting the potential of BC scaffolds for potential use in cell replacement therapies, it first needs to be tested in vivo, and it needs to be confirmed that is does not cause an immune response. There are, however, some promising studies already published that claim that BC is non-immunogenic and has a high biocompatibility [16, 22, 23, 35]. Moreover, future work will also need to explore the delivery strategies as well as the degradation pathways post-implantation in more detail. Importantly, however, the human body does not contain cellulases responsible for the natural degradation of bacterial cellulose, which may help to maintain its mechanical stability and chemical integrity. However, various other oxidative processes and reactive oxygen species might cause partial degradation [32, 33], and thus, further studies on how quickly BC may be degraded in vivo are needed. Recent studies have also focused on the design of various bacterial cellulose composites that might introduce higher stability and a controlled degradation in vivo [32, 33]. All of these developments make bacterial cellulose a highly interesting material for use in neuronal prosthetics, and our study forms an important first step towards the design of fully functional BC-based scaffolds.

It should also be noted that the functionalisation of cellulose is not limited to the proteins used in this study, but can be substituted with any other protein or small molecule of interest providing they contain an available amine group. While in this study we have not characterised the degradation or release rate of the GFs from BC, we are currently working on functionalisation of the cellulose with immunosuppressants using cleavable linkers to enable slow, controllable release. The introduction of such moieties would protect implants from an immune response from the cells during initial transplantation into the brain. In addition, the attachment of proteins and small molecules to the cellulose can also provide signalling functionalities both in vitro and once implanted into the brain, particularly if such molecules are attached using cleavable linkers and thus can diffuse into the brain tissue from the site of implantation [20, 50].

The advantage of this described functionalisation strategy lies in its compatibility with the design of various nano-composite structures. For example, various nanoparticle binding linkers can be inserted to act as anchoring sites for conductive nanostructures such as carbon nanotubes to result in conductive scaffolds that can enable electrical stimulation and restoring signals in electrogenic tissue. The favourable mechanical and physiochemical properties of bacterial cellulose, ease of preparation and biofunctionalisation make it a highly interesting material for use in neuronal implants and regenerative medicine in general. As bionanotechnological tools develop further, design of nanocomposites will provide us with cellulose material that might have enhanced thermal and conductive properties, useful for cell differentiation and in vivo biosensing. We would like to encourage the researchers working in the field to explore this material further to enrich the material toolbox and provide clinically transferable solutions.

## Conclusions

We have shown that bacterial cellulose (BC) scaffolds can be prepared and functionalised to support the differentiation of neuronal stem cells and in such a way be used for the development of a new generation of neuronal implants. To achieve this, we optimised the Beaumont protocol (aqueous one-pot silanisation) [3, 4] to obtain an adaptable strategy that can be used for immobilisation of a wide range of bioactive peptides or small molecules onto BC, which, once proven successful in vivo, may enhance implant efficacy. Using a cell viability assay, we have also shown that there is no measurable toxicity of the scaffold material and no detrimental effects to stem cell differentiation. The proportion of cells that differentiate into neuronal cells once plated on BC was further increased by the functionalisation of the scaffold with laminin, and growth factors BDNF and GDNF.

## Data Availability

The data that support the findings of this study are available from the corresponding author upon reasonable request.

## Abbreviations

BC: Bacterial cellulose
BDNF: Brain-derived neurotrophic factor
GDNF: Glial cell-derived neurotrophic factor
GPTMS: 3-Glycidyloxypropyl-trimethoxysilane growth factors (GFs)
hESCs: Human embryonic stem cells
PD: Parkinson’s disease
PDMS: Polydimethylsiloxane
VM: Ventral midbrain
